# Modular and Versatile Spatial Functionalization of Tissue Engineering Scaffolds through Fiber‐Initiated Controlled Radical Polymerization

**DOI:** 10.1002/adfm.201501277

**Published:** 2015-08-17

**Authors:** Rachael H. Harrison, Joseph A. M. Steele, Robert Chapman, Adam J. Gormley, Lesley W. Chow, Muzamir M. Mahat, Lucia Podhorska, Robert G. Palgrave, David J. Payne, Shehan P. Hettiaratchy, Iain E. Dunlop, Molly M. Stevens

**Affiliations:** ^1^ Department of MaterialsImperial College LondonLondonSW7 2AZUK; ^2^ Institute of Biomedical EngineeringImperial College LondonLondonSW7 2AZUK; ^3^ Department of BioengineeringImperial College LondonLondonSW7 2AZUK; ^4^ Department of Plastic and Reconstructive SurgeryImperial College Healthcare NHS TrustCharing Cross CampusFulham Palace RoadLondonW6 8RFUK; ^5^ Department of ChemistryUniversity College London20 Gordon StreetLondonWC1H 0AJUK

**Keywords:** bilayered materials, antifouling, electrospinning, controlled polymerization, polymer bottlebrushes

## Abstract

Native tissues are typically heterogeneous and hierarchically organized, and generating scaffolds that can mimic these properties is critical for tissue engineering applications. By uniquely combining controlled radical polymerization (CRP), end‐functionalization of polymers, and advanced electrospinning techniques, a modular and versatile approach is introduced to generate scaffolds with spatially organized functionality. Poly‐ε‐caprolactone is end functionalized with either a polymerization‐initiating group or a cell‐binding peptide motif cyclic Arg‐Gly‐Asp‐Ser (cRGDS), and are each sequentially electrospun to produce zonally discrete bilayers within a continuous fiber scaffold. The polymerization‐initiating group is then used to graft an antifouling polymer bottlebrush based on poly(ethylene glycol) from the fiber surface using CRP exclusively within one bilayer of the scaffold. The ability to include additional multifunctionality during CRP is showcased by integrating a biotinylated monomer unit into the polymerization step allowing postmodification of the scaffold with streptavidin‐coupled moieties. These combined processing techniques result in an effective bilayered and dual‐functionality scaffold with a cell‐adhesive surface and an opposing antifouling non‐cell‐adhesive surface in zonally specific regions across the thickness of the scaffold, demonstrated through fluorescent labelling and cell adhesion studies. This modular and versatile approach combines strategies to produce scaffolds with tailorable properties for many applications in tissue engineering and regenerative medicine.

## 1 Introduction

The ability to design surface properties of scaffolds to direct cell and protein binding is key in tissue engineering (TE) and regenerative medicine. Following injury or scaffold implantation there are many scenarios where cell ingrowth or protein fouling onto a tissue or scaffold surface may lead to undesirable outcomes such as problematic scar tissue. This is particularly key at gliding tissue interfaces. Scaffolds that exhibit effective opposing cell‐adhesive and antifouling sides would revolutionize the outcome of injuries and operations at such biological interfaces through their ability to support tissue healing (cell‐adhesive surface) and to reduce cell‐ingrowth and protein adsorption (antifouling surface) in a spatially controlled manner. Injuries or operations to gliding surfaces such as musculoskeletal tissues or involving the peritoneal tissues of the abdomen or pelvis are often complicated with undesirable adhesions. These adhesions are bands of scar tissue that directly result from cellular ingrowth and bridging between previously gliding surfaces resulting in restriction of movement that causes pain and compromised function. Similarly, protein deposition at vascular interfaces such as on artificial blood vessels or at sites of injury can lead to blood clotting that can cause disastrous consequences.

This work presents a modular and versatile scaffold system that has been specifically designed to allow facile modification for specific application. A porous structure manufactured with a processing technique that can add to this versatility is required; this can be achieved with electrospinning. Electrospinning is an ideal technique for producing 3D networks of fibers of tunable size, orientation, composition, and density that mimic the properties of native extracellular matrix (ECM)^1^, ^2^, ^3^ and can generate scaffolds with spatially arranged functionalization through layering of various polymers during electrospinning.^4^, ^5^, ^6^, ^7^, ^8^, ^9^ Developing a controlled method that allows multiple zonally arranged functional groups within a continuous scaffold allows for the production of a hierarchical structure that can modulate cell behavior within each functional zone. Electrospinning and the use of fibers also allows for high density surface functionalization that can dramatically change the surface properties whilst maintaining the spatial control of presentation and fiber morphology.

Controlled radical polymerization (CRP) techniques are facile and versatile methods for providing surface functionalization with a wide range of monomers. These have not been fully exploited in scaffold functionalization and yet are extremely powerful methods for preparing antifouling surfaces. Atom transfer radical polymerization (ATRP) and reversible addition‐fragmentation chain transfer (RAFT) have been used to attach polymers to a variety of surfaces to generate surface‐derived functionality.^10^, ^11^, ^12^, ^13^ These polymerization methods are versatile and offer excellent control of chain length, architecture, reaction kinetics, and they can add a vast array of functionality as a large number of monomers may be incorporated, which dictates the final material properties.^10^, ^14^ In this work we have employed the versatility of surface‐initiated CRP to create an antifouling polymer bottlebrush on one side of an electrospun poly‐*ε*‐caprolactone (PCL) scaffold with a cell‐adhesive peptide on the opposing surface across a diameter of a few hundred micrometers.

The antifouling surface is generated by grafting a high density, antifouling, biocompatible polymer bottlebrush based on poly(ethylene glycol) (PEG) from initiators presented on the PCL scaffold surface. PEG is an antifouling polymer that has been used to mediate cell and protein adhesion to surfaces and has been used in devices approved for implantation into the body.^15^ The ability to incorporate additional (bio)functionalities within the antifouling brushes is showcased with the addition of a biotinylated monomer, which can provide a versatile handle for the *post hoc* addition of various streptavidin‐coupled moieties. To maximize the antifouling ability by creating a dense hydrated polymer layer, we elected to use the “grafting from” approach, whereby the initiating group is attached to the surface and the polymer grows out from it. This avoids the steric hindrance and resultant low density that is found in a “grafting to” approach.^16^ Second, we selected the oligomeric monomer of PEG, poly(ethylene glycol) methyl ether methacrylate (OEGMA) to generate a pOEGMA bottlebrush structure that leads to a vastly higher density of PEG being displayed on the surface for superior performance. Polymer brush growth from electrospun fibers has typically been achieved using ATRP polymerization of a variety of different monomers. In most approaches that are directed towards biomedical applications, the initiating group is incorporated as a post electrospinning modification before polymerization has been undertaken.^17^, ^18^, ^19^, ^20^, ^21^, ^22^ Our strategy offers significant advantages to this by incorporating the initiator as an end‐group to the polymer prior to electrospinning to allow precise control over the spatial position of the functional groups without disrupting the fiber architecture. This approach has been used previously for the polymerization of styrene,^23^ 2‐hydroxyethyl methacrylate,^16^ and *N,N*‐isopropylacrylamide,^24^ but not in a biomedical application. The selection of the initiating group and electrospinning arrangement further allows surface enrichment of the fibers with initiating groups through electrostatic attraction.^23^ Second, a modified form of ATRP, namely, activators regenerated by electron transfer atom transfer radical polymerization (ARGET ATRP), was employed that avoids the high concentrations of potentially toxic transition metal catalyst and organic solvents used in conventional ATRP.^25^ This is critical for scaffolds designed for biomedical use as high levels of contamination can be difficult to thoroughly remove from a bulk scaffold. Finally, in contrast to conventional ATRP, ARGET is less sensitive to small amounts of oxygen contamination, offering increased ease of use.^26^ To our knowledge this is the first use of controlled radical polymerization to polymerize pOEGMA from a PCL surface.

Our approach of pre‐functionalizing PCL with end‐functional groups before electrospinning offers a facile and versatile method for maintaining scaffold architecture, functionality, and material properties whilst having precise control over the spatial location and molecular weight of the grafted polymer. It also allows control over the density of functional groups by simply changing the concentration of the PCL conjugates. Specifically, we modified one batch of PCL with the initiating group for polymerization and a separate batch with the canonical adhesion peptide sequence Arg‐Gly‐Asp‐Ser (RGDS). The polymer conjugates were sequentially electrospun as a bilayer to achieve spatial control of the functionality and surface properties. This work builds from our recent work using sequential electrospinning to form opposing gradients of two different peptides in a PCL scaffold, which directed the specific binding and spatial organization of biopolymers (glycosaminoglycans, GAG) within the scaffold.^27^ These techniques provide a new and versatile platform for the preparation of multi‐functional TE scaffolds to address unmet clinical need in orthopedic, plastic, reconstructive, and general surgery.

## 2 Results and Discussion

### 2.1 Production of Polymer Bottlebrush through Surface‐Initiated Polymerization

ARGET ATRP reaction kinetics were first established and optimized in solution; polymerizations were conducted in water/IPA (1:1 v/v) in order to prevent dissolution of the PCL. A molar ratio of 150:1:1:0.15 OEGMA : initiator : Cu(II)Cl_2_ : sodium ascorbate at 30°C resulted in reproducible >70% conversion within 2 h. Good control was achieved as evidenced by pseudo first‐order kinetics and the low molecular weight dispersity of the polymers (*M*_w_/*M*_n_ < 1.3).

**Figure 1:**
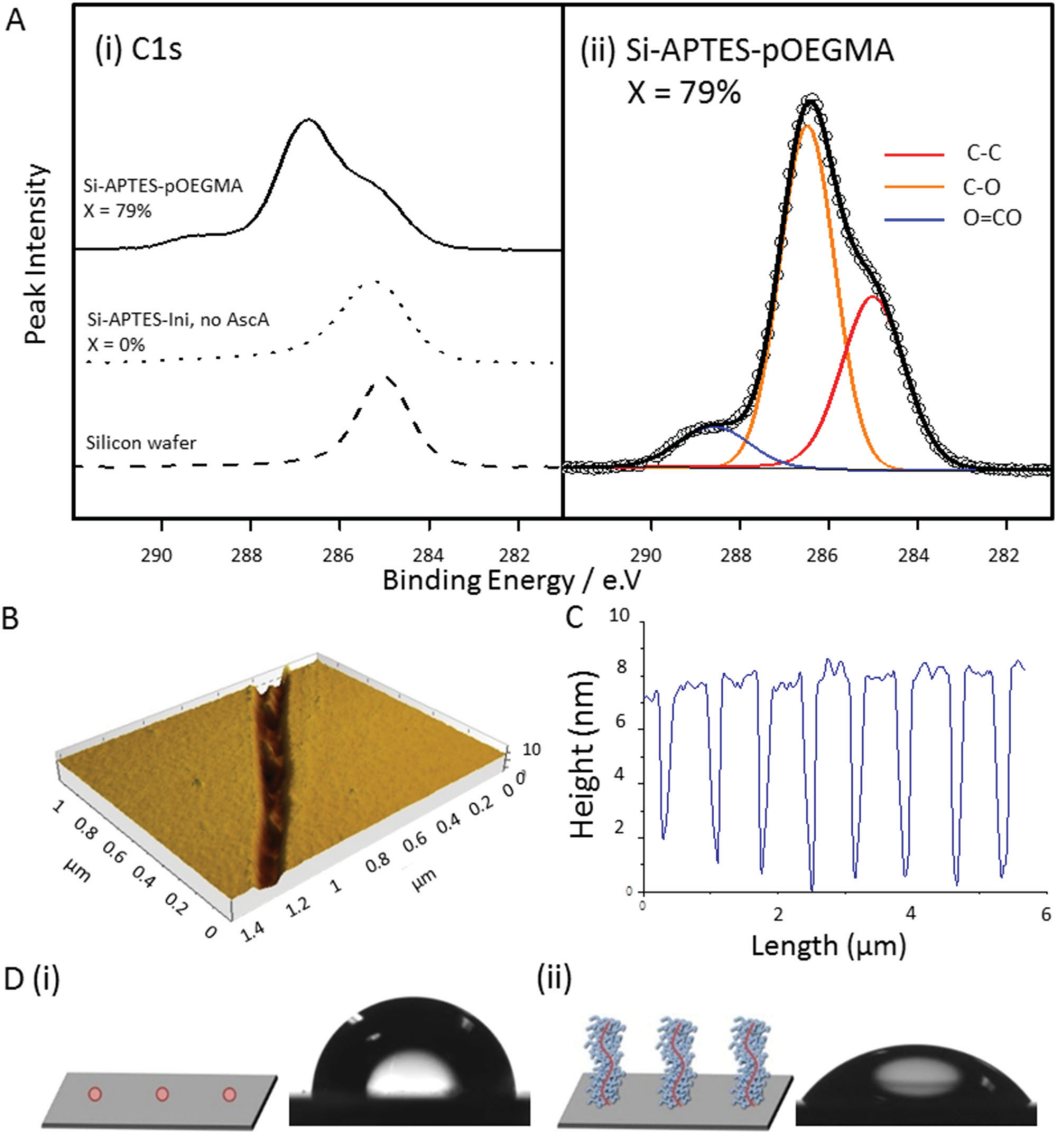
Demonstration and characterization of surface‐initiated polymer brush growth from functionalized 2D silicon surfaces. A) XPS analysis of pOEGMA grafting from a silicon wafer functionalized with APTES‐Ini with controls (dashed bottom trace), silicon functionalized with APTES‐Ini that underwent polymerization with no reducing agent, ascorbic acid (AScA, dotted middle trace), and pOEMGA grafting from a silicon wafer (top trace, left). Conversion by ^1^H‐NMR (X) is included above each trace. Si‐APTES‐pOEGMA sample with the C1s peaks fitted (right). B) AFM scratch test and C) representative profile of pOEGMA grafted from silicon wafers. D) Water contact angle measurement of a silicon wafer functionalized with (i) APTES‐Ini and (ii) following grafting of pOEGMA.

Surface‐initiated ARGET ATRP of pOEGMA was successfully performed from 2D silicon surfaces, silicon wafers, optimized and characterized, before progressing to 3D electrospun scaffolds to ensure reproducible results. The initiating group α‐bromoisobutyryl bromide (BiBB) was attached to the surfaces using 3‐(aminopropyl)triethoxysilane (APTES) before pOEGMA was grafted from the wafer. A free sacrificial initiator, ethyl‐α‐bromoisobutyrate (EBiB), was used in solution to aid control of the polymerization and to allow analysis of the free polymer as a surrogate for the surface bound polymer; this has been shown to be a reliable tool for controlling the *M*_n_ and *M*_w_/*M*_n_ for the polymers grown from surfaces within the same reaction vessel.^28^ Ellipsometry and atomic force microscopy (AFM) confirmed the presence of a polymer brush layer with a dry thickness of ≈6.2 nm (±0.038 nm, MSE 3.548) (**Figure** **1**B,C and Figure S1, Supporting Information), and X‐ray photoelectron spectroscopy (XPS) confirmed this layer to be organic with the expected changes in the ratio of the C—O bond (Table S1, Supporting Information). Further evidence for the successful polymerization of OEGMA from the surface is given by the increased wettability after polymerization with a change in water contact angle from 63.6° ± 2.3° on Si‐APTES‐Ini functionalized wafers to 36.3° ± 5.9° (Figure 1D).

### 2.2 Surface‐Initiated Polymerization from 3D Electrospun Fibers

**Figure 2:**
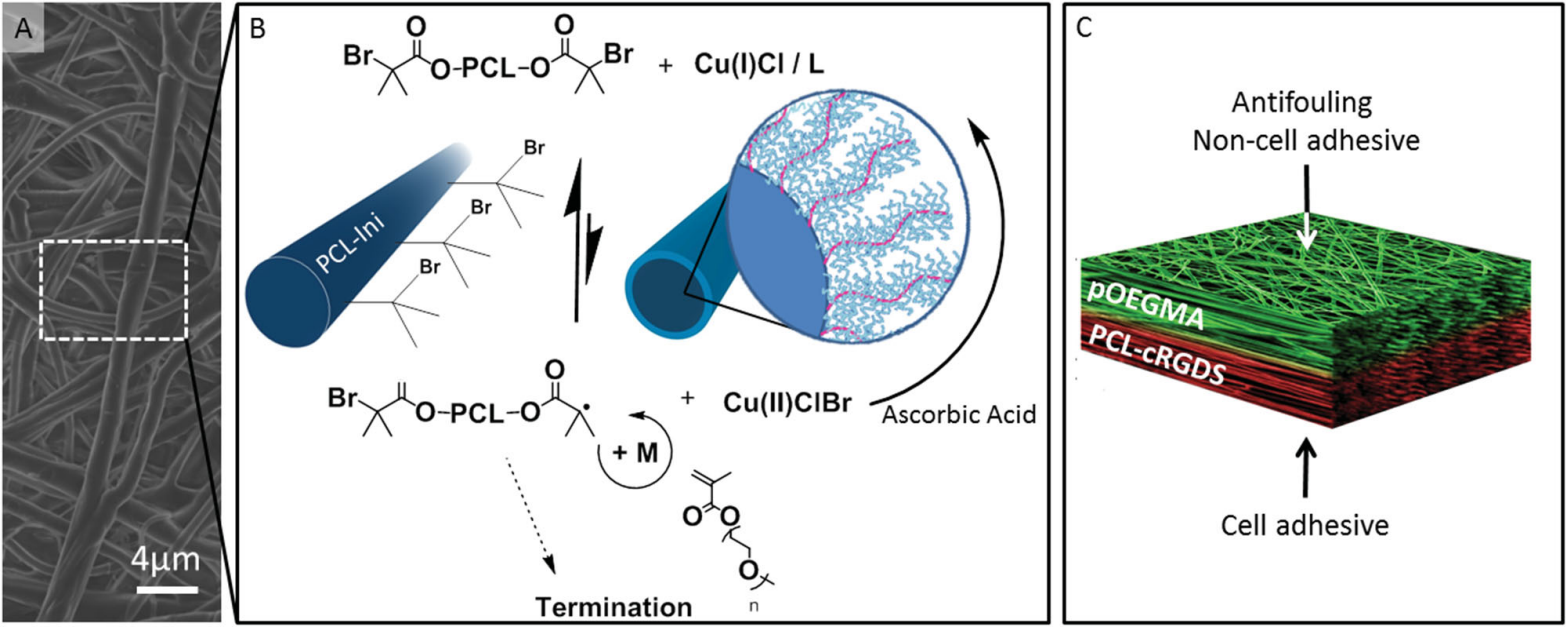
Grafting of pOEGMA bottlebrushes from prefunctionalized electrospun scaffolds to create an antifouling, non‐cell‐adhesive surface as part of a dual functional scaffold. A) Representative SEM micrograph of electrospun PCL‐pOEGMA fibres. B) ARGET ATRP reaction scheme for polymerization of pOEGMA from the PCL‐Ini fibers with inset schematic images of PCL‐Ini following electrospinning (left) and following polymerization of pOEGMA from the fiber surface (right). C) Schematic outlining the bifunctional scaffold structure produced using layered electrospinning with postprocessing polymerization to create an antifouling PCL‐pOEGMA surface with an opposing cell binding PCL‐cRGDS surface.

To undertake surface‐initiated polymerization from 3D electrospun fibrous scaffolds we commenced by modifying a PCL‐diol (*M*_w_ 14 000 Da) with the initiating group (BiBB) to produce a polymerization initiating region at either end of the PCL polymer chain (PCL‐Ini). PCL was selected to form the bulk of the scaffold due to its bioresorbability, good handling properties, electrospun fiber morphology, suitable degradation rate, ease of chemical modification, and its current use in Food and Drug Administration (FDA)‐approved devices.^29^ To ensure that the initiating region of the PCL conjugates was presented on the fiber surface, we set up the electrospinner with the cathode at the spinnerette to convey a positive charge to the surface of the polymer solution. The alkyl‐bromide group present within BiBB can become electronegative due to its polarity,^23^ dragging it electrostatically to the surface of the polymer solution stream and leading to surface enrichment of the initiating group on the fiber. The PCL‐Ini was subsequently electrospun in combination with a high molecular weight PCL to form functionalized fibrous scaffolds which were then imaged by scanning electron microscopy (SEM) to validate consistent fiber morphology (**Figure** **2**A). The addition of up to 17% (w/w) of the PCL‐Ini did not significantly alter the electrospinning process or fiber formation (Figure S2, Supporting Information). pOEGMA bottlebrushes were grafted from the electrospun fibers using the parameters optimized in the 2D silicon system (Figure 2B). As with the 2D silicon surfaces, a sacrificial initiator was included in order to target a degree of polymerization (DP) of 150. A typical polymerization achieved a ≈75% conversion (by ^1^H‐NMR) and *M*_n_ 45 000, *M*_w_ 53 000 with a dispersity (*M*_w_/*M*_n_) of 1.18 (from size exclusion chromatography, SEC, analysis of the free polymer).

**Figure 3:**
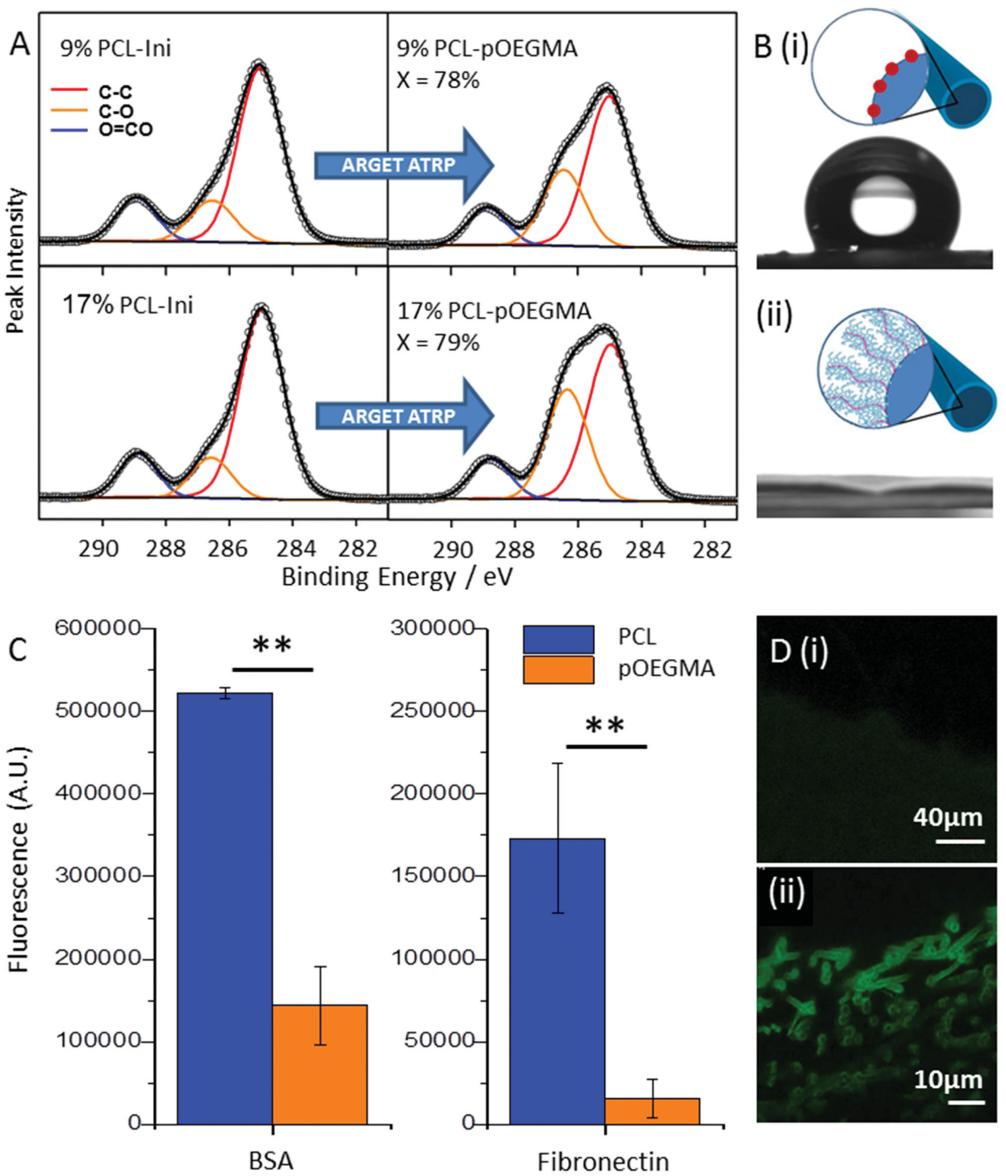
Demonstration and characterization of surface‐initiated polymer brush growth from functionalized 3D electrospun scaffolds. A) High resolution C1s core‐level spectra of pOEGMA grafting from electrospun scaffolds with 9% and 17% (w/w) PCL‐Ini before (left) and after (right) grafting of pOEGMA. Conversion by ^1^H‐NMR (X) is inset. B) Water contact angle measurement of (i) electrospun PCL/PCL‐Ini and (ii) PCL‐pOEGMA with inset schematics. C) Antifouling ability of PCL and PCL‐pOEGMA electrospun scaffolds was compared using fluorescently labelled proteins and GAGs, expressed as μg cm^−2^ of electrospun scaffold, **p < 0.005. D) PCL (i) and PCL‐p(OEGMA‐*co*‐biotin) (ii) fibers labeled with fluorescein‐streptavidin and imaged using confocal microscopy.

XPS confirmed successful grafting of pOEGMA from functionalized electrospun scaffolds as a large increase in C—O signal is observed following polymerization for both the 17% PCL‐Ini (w/w) and the 9% PCL‐Ini (w/w) (**Figure** **3**A). This is in contrast to the control scaffold lacking in initiating groups that shows minimal increase in the C—O signal confirming successful covalent attachment of polymer to the scaffolds through the initiating groups (Table S1, Supporting Information). A dramatic change in the water contract angle adds further evidence for successful pOEGMA grafting. Hydrophobic electrospun PCL/PCL‐Ini scaffold surfaces, with contact angles of 113.5° ± 7.8°, become hydrophilic after polymerization, with the water droplet immediately completely wetting the surface (Figure 3B).

To visualize the polymer brush and to demonstrate the versatility of the system we included a biotinylated monomer unit (biotinylated PEG monomer **3**, Supporting Information) that could be co‐polymerized into the bottlebrush. The resultant random co‐polymer of PCL‐pOEGMA‐*co*‐biotinylated PEG (PCL‐p(OEGMA‐*co*‐biotin)) allows labeling with streptavidin‐conjugated probes. Following histological sectioning of the scaffolds, labelling with fluorescein‐streptavidin, and imaging with confocal microscopy, the fluorescent signal was clearly visualized on the electrospun fibers demonstrating that the polymer brush is grafted from the fiber surface (Figure 3D). Histological sections of the scaffolds imaged with wide field fluorescent microscopy further demonstrated the polymer brush evenly distributed throughout the cross section of the scaffold (Figure S3, Supporting Information) and further imaging of electrospun scaffolds following grafting of PCL‐p(OEGMA‐*co*‐biotin) confirmed the covalent attachment of the pOEGMA to the fiber surfaces. A control scaffold of electrospun PCL without the initiating group was present in the same reaction vessel as PCL‐Ini (17% w/w) scaffolds and demonstrated no fluorescent signal following washing, and incubation with fluorescein‐streptavidin. The successful surface grafting of pOEGMA is further supported by the difference observed in water contact angle between electrospun scaffolds with and without initiating groups that underwent polymerization. The control scaffolds remained hydrophobic while the pOEGMA functionalized scaffolds were highly hydrophilic (Figure S4, Supporting Information).

### 2.3 pOEGMA Surface Functionalization for Antifouling and Prevention of Cell Adhesion

pOEGMA is known to have antifouling properties and we looked to demonstrate this property from the functionalized scaffold.^30^ 17% (w/w) PCL‐pOEGMA scaffolds having achieved a conversion of >72% within the same reaction vessel were tested for antifouling and resistance to cell adhesion. pOEGMA grafted scaffolds demonstrated excellent antifouling ability and resistance to common ECM protein absorption when compared to PCL scaffolds. This was established using a panel of fluorescently labelled proteins and GAGs. These biomolecules were chosen as they represent a range of molecular weights, charge, and hydrophilicity, and several are known to modulate the binding and activity of other biomolecules such as growth factors. Adsorption of these molecules to a surface would likely lead to increased biomolecule deposition and ultimately, cell adhesion. pOEGMA scaffolds dramatically outperformed native PCL for both bovine serum albumin (BSA) and fibronectin showing a 3.6 fold decrease in binding for BSA and greater than ten fold decrease for fibronectin (Figure 3C). Relative to BSA, adsorption of hyaluronic acid (HA), heparin, and chondroitin sulphate (CS) for both PCL and pOEGMA scaffolds was negligible.

**Figure 4:**
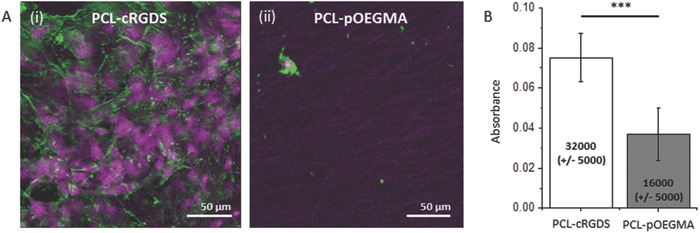
Cell‐adhesive and non‐cell‐adhesive properties of functionalized electrospun scaffolds. A) Representative confocal microscopy images of bovine tenocytes cultured for 7 d on electropun PCL‐cRGDS (i) and PCL‐pOEGMA scaffolds (ii). Cell nuclei stained with draq5 (purple) and actin with phalloidin (green). B) Metabolic activity of bovine tenocytes cultured on scaffolds for 7 d was assessed by MTT assay. Estimated cell number is stated for each bar. *** Significant difference (*p* < 0.0001), error bars represent standard deviation.

**Figure 5:**
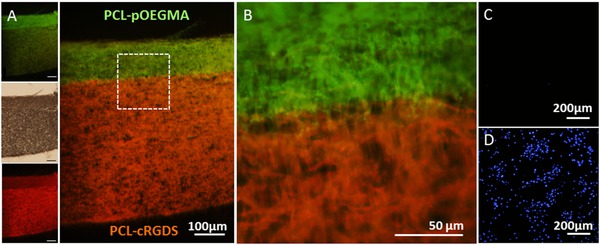
Dual functionality scaffolds demonstrated by fluorescent labelling of functionalities and cell adhesion. Fluorescence microscopy images of cross sections of bi‐functional scaffolds formed with opposing PCL‐Ini and PCL‐cRGDS surfaces. Post‐processing polymerization was used to produce a PCL‐p(OEGMA‐*co*‐biotin) surface. A,B) Overlaid fluorescence images of histological cross‐sections labelled with fluorescein‐streptavidin (green) on the p(OEGMA‐*co*‐biotin) and with Cy5 (red) for the PCL‐cRGDS showing well defined spatial resolution. Insets (left) show the brightfield and single channel fluorescence images with 100 μm scale bars. C,D) Bovine tenocytes were seeded on fresh scaffolds and cultured for 7 d before being stained with DAPI (blue) for cell nuclei and imaged with fluorescent microscopy. The PCL‐pOEGMA surface is shown in the upper image (C) and the opposing PCL‐cRGDS surface is shown in the lower image (D).

In preparation for the creation of the dual functionality scaffold we prepared a cell adhesive PCL to compare to the PCL‐pOEGMA. We selected the canonical peptide motif RGDS sequence as a model cell‐adhesive biomolecule that promotes cell adhesion through integrin binding.^31^ Fibroblasts, of which tenocytes are a specialized form, are known to bind to RGDS.^32^ PCL was conjugated to a cyclized form of the RGDS (cRGDS), the natural presentation of the ligand in fibronectin.^33^ PCL was conjugated with the cRGDS (Supporting Information) and electrospun into a scaffold using the standardized protocol. Bovine tenocytes were seeded onto both the PCL‐cRGDS and PCL‐pOEGMA scaffolds and cultured for 7 d to assess how the different surfaces supported cell adhesion and survival. Tenocytes seeded on the PCL‐cRGDS scaffolds adhered well, formed a confluent cell layer, and exhibited spread morphology as demonstrated by confocal imaging of the scaffold surface. Conversely, the cells seeded on the PCL‐pOEGMA scaffolds were few in number and those found were more rounded in morphology, indicating poor attachment (**Figure** **4**A). The lack of robust attachment to the scaffold, as indicated by the rounded morphology, may have resulted in the surface cells being washed away while the remaining cells were trapped within the fibrous structure. These observations were compared for the whole scaffolds using a colorimetric assay for cellular metabolic activity based on the reduction of the tetrazolium dye 3‐(4,5‐dimethylthiazol‐2‐yl)‐2,5‐diphenyltetrazolium bromide (MTT) to approximate the relative number of cells (Supporting Information). A large reduction *(p* < 0.0001) in overall metabolic activity was observed at day 7 between PCL‐cRGDS and PCL‐pOEGMA scaffolds implying a reduced cell number on the PCL‐pOEGMA scaffolds (Figure 4B). The estimated cell numbers are somewhat higher than the appearance of the scaffolds by confocal microscopy would suggest. This reflects the presence of a small number of rounded cellular aggregates on the PCL‐pOEGMA surface, indicative of preferential cell–cell interactions over cell–surface interactions, in contrast to the densely populated spread cell morphology seen on the PCL‐cRGDS surface. Together, the estimated cell numbers and confocal micro­scopy findings show consistently different cellular adhesion between the PCL‐pOEGMA and PCL‐cRGDS surfaces. This is preserved in the bi‐functional scaffold, as evidenced by fluorescence microscopy, in accordance with our design (**Figure** **5**C,D).

### 2.4 Spatial Control of Polymer Brush Leading to a Dual Functionality Scaffold

Having demonstrated the antifouling property of the PCL‐pOEGMA surface and cell‐adhesive property of the PCL‐cRGDS surface, we progressed to immobilizing them within a single construct. We sequentially electrospun the two functionalized polymers, PCL‐Ini and PCL‐cRGDS, to produce discrete sections within the same electrospun construct. To confirm the presence and spatial location of the functionalized PCL, we used specific fluorescent labels to tag corresponding functional groups within the pOEGMA or cRGDS sections. The PCL‐pOEGMA side included the biotinylated monomer previously described, to produce a PCL‐p(OEGMA‐*co*‐biotin) brush that could be labeled with streptavidin‐fluorescein. To visualize the cRGDS, we modified a Cy5 dye with an amine, which can react with the carboxylic acids found only on the aspartic acid (D) residue side chains exposed on the PCL‐cRGDS fibers (Figure S13, Supporting Information). Histological sections of the dual functional scaffolds were labeled to illustrate these discrete fiber locations and show successful spatial control of the different functionalities within the substance of a single electrospun construct (Figure 5A,B).

To further interrogate the dual functionality scaffolds, bovine tenocytes were seeded on both surfaces of the scaffolds and cultured for 7 d. The scaffolds were then stained with 4′,6‐diamidino‐2‐phenylindole (DAPI) to label the cell nuclei on both sides of each scaffold and fluorescent imaging was performed by imaging one side, before turning it over and imaging the opposite side. The desirable functionality of the opposing surfaces is preserved despite the bilayer processing. The PCL‐cRGDS surface supports a high cell density whereas very few cells are seen on the opposing pOEGMA surface (Figure 5C,D). Excellent spatial control of functionalized polymers is demonstrated by the sharp transition of layers seen with fluorescent labelling. Sequential electrospinning of end‐group functionalized polymers is shown to be a highly effective method for spatial control within a construct. This could be used to generate more complex architecture including multilayering.

## 3 Conclusion

In conclusion, an effective dual functionality scaffold with a cell adhesive surface and an opposing antifouling, non‐cell adhesive surface has been successfully produced using a combination of end functionalization of PCL with a polymerization initiating group (BiBB) and a cell binding motif (cRGDS) that may be sequentially electrospun to produce a scaffold with zonally arranged functional groups. Post‐processing with ARGET ATRP is a facile and highly versatile method to produce a surface‐initiated pOEGMA bottlebrush that elicits a high performance antifouling and cell resistant coating. We have demonstrated the versatility of the polymerization for the addition of multifunctionality through the use of a biotinylated mono­mer unit in a single processing step. This modular and versatile approach could be used as a platform scaffold for multiple applications in tissue engineering.

## 4 Experimental Section

All chemical reagents were purchased from Sigma Aldrich (UK) and deuterated solvents for ^1^H‐NMR from Merck (Darmstadt, Germany) unless specifically noted.

*ARGET ATRP of OEGMA in Solution and from Surfaces*: pOEGMA bottlebrushes were produced in solution and from 2D silicon and 3D electrospun functionalized surfaces. When producing brushes from surfaces, a sacrificial initiator, ethyl 2‐bromoisobutyrate (EBiB), was used in solution to enhance control of the polymerization and allow for surrogate characterization of the polymerization on the surface.^14^, ^34^ The amount of initiator present on the 2D silicon and 3D electrospun surfaces was estimated and if greater than 10% of the sacrificial initiator mass, the mass of sacrificial initiator was reduced proportionally to maintain the reaction conditions.

In a typical experiment OEGMA (340 mg, 0.7 mmol, with a *M*_w_ of the poly(ethylene glycol unit) of 400 Da, Polysciences, Germany), activated by the removal of inhibitors, was introduced into a round bottom flask with copper (II) chloride (Cu(II)Cl_2_, 0.64 mg, 0.0047 mmol), tris[(2‐pyridyl)methyl]amine (TPMA, 1.37 mg, 0.0047 mmol), and EBiB (0.92 mg, 0.0047 mmol) in 3 mL of 50:50 isopropyl alcohol (IPA)/H_2_O. Following through mixing 2 mL was transferred to a test tube that was empty or containing functionalized 2D silicon or 3D electrospun surfaces. It was sealed, introduced into an ice bath, and degassed with argon for 20 min before ascorbic acid (AscA 0.08 mg, 0.00047 mmol) was added from a degassed solution using a gas tight syringe. The vessel was transferred to a pre‐warmed heat block at 30 °C and allowed to react for 2 h at which time the reaction was quenched by bubbling oxygen through the reaction mixture. Conversion was calculated by ^1^H‐NMR (400 Hz, *d*4‐MeOD) and molecular weights determined by SEC using a GPCMax VE 2001 (Viscotek). The SEC was run with an eluent of *N,N*‐dimethylformamide (DMF) with 0.075% (w/v) at a flow rate of 0.7 mL min^−1^ over two Polymer Standards Service (PSS) Gram DMF columns at 35 °C. Molecular weights were determined relative to pMMA standards (Agilent Technologies, UK) without correction. Prior to the measurements the copper was removed using heavy metal chelating beads (Cuprisorb, Fish Fish Fish, UK) and filtered through a 0.22 μm syringe mounted polytetrafluoroethylene filter. Scaffolds and silicon wafers functionalized with polymer brushes were removed from solutions and thoroughly rinsed three times in 100% ethanol before washing in Milli‐Q H_2_O for 24 h with three intermittent water changes. Any remaining copper was removed through the addition of Cuprisorb heavy metal chelating beads in the final washing step before drying in a vacuum desiccator at room temperature.

*Functionalization of Silicon Wafers with APTES and BiBB for 2D Silicon Polymerization*: P‐doped silicon wafers (University Wafer, Boston, USA) were prepared for functionalization with sonication in acetone for 3 min, rinsing with Milli‐Q H_2_O and immersion in piranha solution (1:3, hydrogen peroxide: concentrated sulfuric acid) for 1 h, rinsing twice with Milli‐Q H_2_O, and drying under a stream of nitrogen. APTES was deposited on the surface of the silicon wafers using vapor deposition using a modified protocol from the literature; cleaned silicon wafers were laid in a glass petri dish and a glass vial containing 10 mL of anhydrous hexane and 0.25 mL of APTES was placed in the center of the dish.^35^ The petri dish was placed in a desiccator to which a vacuum was applied and maintained for 90 min. Wafers were subsequently removed and inserted into test tubes, sealed with septa and parafilm, degassed with nitrogen and to each a degassed solution of 5 mL of anhydrous hexane, 0.125 mL of BiBB, and 0.165 mL of anhydrous triethylamine (TEA) was introduced and allowed to react at room temperature for 1 h. Following this the wafers were removed and washed with hexane, ethanol and Milli‐Q H_2_O and then dried under a stream of nitrogen. Wafers were stored in a vacuum desiccator and protected from light until used.

*Water Contact Angle Measurement*: Water contact angles on 2D silicon wafer surfaces and electrospun scaffolds were measured with a Kr˝uss Easy Drop DSA 100 (Hamburg, Germany) and the associated DSA1 v 1.9 software, a drop size of 7 μL, and at room temperature.

*Brush Thickness Measurements with AFM and Spectroscopic Ellipsometry*: Dry thickness measurements were undertaken with AFM and ellipsometry on the same 2D silicon samples to establish the thickness of the grafted pOEGMA layer. AFM measurements were taken using an Agilent Technologies 5500 atomic force microscope with a silicon probe, tip radius <10 nm, force constant 40 nN m^−1^. A scratch test was performed whereby the AFM probe tip was moved towards the sample, contact was made, and the force increased until the underlying silicon wafer was contacted. The tip was then moved laterally to create a full‐thickness scratch. The AFM was then used in tapping mode to create a depth profile across the scratch area. Using the associated software (Pico Image) thickness measurements of the polymer layer were made.

Ellipsometry measurements were performed at room temperature using a SOPRA GESP 5 variable angle spectroscopic ellipsometer. The data were recorded through incidence angles of 65°–75° with respect to the substrate normal, across a wavelength range from 900–1600 nm (20 nm steps). Cauchy model fits were used to analyze the ellipsometric data. Good agreement was found between the ellipsometrically deduced brush thicknesses and AFM.

*Production of Functionalized Electrospun Scaffolds and Fiber‐Initiated Controlled Radical Polymerization–Synthesis of PCL‐Ini**1***: PCL‐diol (*M*_w_ 14 000 Da) was functionalized with 2‐bromoisobutyryl bromide (BiBB) using a protocol adapted from the literature to produce PCL‐Ini**1**.^24^ In a typical run, 5 g of 14 kDa PCL‐diol was introduced to 200 mL of anhydrous tetrahydrofuran in a round bottom flask and stirred at room temperature in a sealed nitrogen atmosphere. Following dissolution of the PCL, 2.9 mL TEA and 50 mg of 4‐dimethylaminopyridine were added and the vessel immersed in an ice bath. After 15 min of cooling, 265 μL of BiBB was introduced dropwise, the ice bath removed, and the reaction stirred overnight at room temperature. The mixture was filtered, and the filtrate was collected, reduced through vacuum rotary evaporation, and precipitated into cold diethyl ether. The precipitate was isolated by vacuum filtration and dried. The product was confirmed using ^1^H‐NMR (400 MHz, deuterated CDCl_3_) and was stored until needed in a vacuum desiccator, protected from light. ^1^H NMR (400 MHz, CDCl_3_) *δ* ppm: 4.24 − 4.20 (m, 4H) 4.05 (*t*, *J* = 6.7 Hz, 240H), 3.78 − 3.71 (m, 4H), 2.30 (*t*, *J* = 7.5 Hz, 244H), 1.92 (s, 0H), 1.73 − 1.55 (m, 480H), 1.46 − 1.32 (m, 252H) (see Figure S5, Supporting Information, for peak assignments).

*Electrospinning PCL and PCL Conjugates*: Two different PCL‐Ini:PCL ratios were electrospun into scaffolds; 12 mg mL^−1^ (9% w/w) PCL‐Ini or 24 mg mL^−1^ (17% w/w) PCL‐Ini was added to 12% (w/v) PCL (*M*_n_ 70 000–90 000 Da) in 1,1,1,3,3,3‐hexafluoro‐2‐propanol (HFIP) and mixed overnight. Solutions were transferred to plastic syringes, loaded onto a programmable syringe pump (Kd Scientific, UK) and extruded at a rate of 2 mL h^−1^ through a blunt 18‐gauge stainless steel needle charged with +16 kV (Glassman, Bramley, Hampshire, UK). The needle was placed at distance of 11 cm from a grounded 10 × 10 cm plate for small master scaffolds or a mandrel rotating at 0.33 m s^−1^ for large master scaffolds, each coated with aluminum foil. No difference was seen in fiber alignment or morphology between collectors. All scaffolds were electrospun under the same conditions, stored in a vacuum desiccator, and protected from light until needed.

*XPS Analysis of the 2D Silicon and 3D Electrospun Polymer Brush Functionalized Surfaces*: XPS was used to characterize the surface of both the 2D silicon and 3D electrospun samples. The spectra were measured using a Thermo Fisher K‐Alpha XPS System (Thermo Fisher Scientific Inc.) with a monochromatic Al‐Kα (energy = 1486.71 eV) X‐ray source. Samples were positioned at the electron take‐off angle normal to the surface with respect to the analyzer. Survey spectra were measured over a range of 0–1400 eV and recorded for each sample, then followed by high resolution spectra for C1s and O1s. A low energy electron/ion flood gun was used to ensure effective surface charge compensation. XPS spectra were calibrated to the adventitious C1s signal (285.0 eV). Curve fitting was carried out using Thermo Avantage Software (v. 5.948) using a Shirley background. Peak areas were normalized within Thermo Avantage using atomic sensitivity factors for the Al Kα anode (“AlWagner” library)^36^ and from these areas the carbon composition and elemental ratios were determined.

*Modification of the Polymerization Protocol for Use with the Biotinoylated PEG Monomer 3*: For samples requiring fluorescent labeling, minor modifications were needed to mitigate any possible chelation of the copper catalyst by additional groups in the reaction mixture. The reaction was prepared as previously outlined but with 5 mol% of the OEGMA replaced with biotinylated PEG monomer 3 (Supporting Information), and the reaction was left to proceed overnight. Samples were washed and prepared as previously described.

*Histological Sectioning, Labeling, and Fluorescent Imaging of the Scaffolds*: Scaffolds for histological section and analysis were embedded in polyester wax (VWR, UK) in a method modified from Steedman et al.^37^ Scaffolds were embedded following incubation in a series of wax solutions maintained at 42 °C, first 30 min in 1:1 (v/v) polyester wax and ethanol, followed by two rounds of pure polyester wax for 30 min and 1 h, respectively. The scaffolds were then embedded vertically in polyester wax and allowed to set. Sections were cut at 10 μm onto untreated glass slides, dried, dewaxed with ethanol, and affixed at either end with a drop of inert adhesive. Sections were blocked with 1% (w/w) BSA and 0.1% (w/v) tween 30 in phosphate buffered saline (PBS) for 30 min, stained for 15 min with fluorescein‐streptavidin (Vector Labs, UK) diluted to 1 μg mL^−1^ in 1% (w/w) BSA in PBS at pH 8.4, and washed three times in PBS. Slides were coverslipped for confocal microscopy with FluorSave fluorescent mounting media (Millipore, UK). Standard fluorescent imaging was performed on a Leica inverted optical microscope fitted with an Olympus DP70 digital camera. Confocal imaging was performed on a Leica SP5 inverted confocal microscope and images processed using GIMP 2.1.

*Measurement of Protein Adsorption onto PCL and pOEGMA Scaffolds*: Fluorescently labeled rhodamine‐heparin (rhod‐hep, *M*_W_ 18 kDa), rhodamine‐CS (rhod‐CS, *M*_W_ 50 kDa), and fluorescein‐HA (fluor‐HA, *M*_W_ 1500 kDa) were purchased (Creative PEGWorks, Winston Salem, USA). Rhodamine‐fibronectin (rhod‐fib) and rhotamine‐BSA (rhod‐BSA) were synthesized prior to experimentation (Supporting Information). Stock solutions of labeled protein were 50 μg mL^−1^ in PBS for all proteins with the exception of rhod‐BSA which was 10 μg mL^−1^ in PBS. Circular PCL‐Ini scaffolds, 6 mm in diameter, with and without pOEGMA brush functionalization were immersed in 70% (v/v) ethanol and washed with PBS three times to ensure uniform hydration. Excess liquid was removed and the scaffolds introduced into high return 1.5 mL centrifuge tubes for incubation with 200 μL protein solution (test samples) or PBS (control) at 37 °C for 18 h. Scaffolds were then washed in PBS in 28 mL light protected glass vials overnight to remove any unbound protein. The fluorescent signal in the scaffolds was then quantified on a Perkin Elmer Envision multimode detector (Germany) at an excitation wavelength of 550 nm and emission of 580 nm for rhodamine labeled proteins and at an excitation wavelength of 490 nm and emission of 520 nm for fluorescein labeled proteins.

*Cell Adhesion Testing of PCL‐cRGDS and PCL‐pOEGMA Scaffolds*: Bovine tenocytes were isolated through primary cell culture from three independent animals all of which were a maximum of 2 years in age (Supporting Information). Cell adhesion experiments were performed using a protocol modified from the literature.^38^ In brief, 24‐well plates were coated with two‐component silicon elastomer (Sygard 184, Dow Corning) prepared in a 10:1 ratio and cured for 48 h. Each independent experiment utilized seven PCL‐pOEGMA scaffolds of 6 mm diameter, that had previously been functionalized with a pOEGMA brush and seven 6 mm diameter PCL‐cRGDS scaffolds. Scaffolds and stainless steel insect pins (0.15 mm, Watkins and Doncaster, UK) were immersed into 70% (v/v) ethanol for 15 min before washing in sterile PBS supplemented with 1% (w/v) anti/anti three times. The silicone‐coated well plate was thoroughly sprayed with 70% (v/v) ethanol before a scaffold was inserted into each well and fixed with an insect pin through the center of the scaffold. A row of empty wells was left as a control. The plate and scaffolds were sterilized under UV light in the cell culture hood for 8 h. Scaffolds were washed with sterile PBS immediately before cell seeding.

Bovine tenocytes were prepared in a single cell suspension at a concentration of 5 × 10^5^ cells mL^−1^ from which 50 μL (2.5 × 10^4^ cells) were seeded onto each scaffold. After allowing 2 h for cell attachment at 37 °C, 5% CO_2_, and 100% relative humidity, a further 1 mL of normal growth media (NGM) was gently added to each well. Seeded scaffolds were cultured for 7 d, with the media replaced after the fourth day. On the seventh day the media was aspirated from the wells and the scaffolds were washed with sterile PBS. Two scaffolds of each type were prepared for imaging, the remaining five scaffolds of each type were used for quantification of cellular metabolism using an MTT assay. This was compared to a standard curve produced from a cell ladder to estimate cell number.

Scaffolds reserved for imaging were fixed in 4% (w/v) paraformaldehyde (PFA) for 15 min, washed, and stored at 4 °C in PBS until used. Prior to imaging, scaffolds were blocked in 1% (w/v) BSA and 0.1% (w/v) tween 20 in PBS for 30 min. A solution of 5 × 10^−6^ m draq5 (Thermo Scientific, UK) to stain cell nuclei and phalloidin (Alexa Fluor 488, diluted 1:400, Life Technologies) to stain actin was diluted in 1% (w/v) BSA in PBS and was incubated with the scaffolds under light protection for 20 min before being washed with further PBS. Samples were imaged in PBS by inverted confocal microscopy as previously described.

*Production of Dual Functionality Scaffolds and Characterization*: Dual functionality scaffolds were fabricated as a continuous scaffold using layered electrospinning. Two separate solutions of functionalized PCL were prepared in HFIP as above with the addition of 1 mg mL^−1^ PCL‐cRGDS to one, and 17% (w/w) PCL‐Ini to the other. These were sequentially electrospun in accordance with the protocol described above. The PCL‐cRGDS solution (2 mL) was electrospun first, followed by PCL‐Ini solution. This was allowed to run for a further 30 min to ensure coverage of the scaffold. Post‐processing polymerization of pOEGMA and pOEGMA‐*co*‐biotin was performed as described above.

*Sectioning, Labeling, and Imaging of Dual Functionality Scaffolds*: Dual functionality scaffolds with a PCL‐p(OEGMA‐*co*‐biotin) surface were embedded in polyester wax and blocked out in a vertical orientation before sectioning at 10 μm onto glass slides, and dewaxed with ethanol as previously described. The scaffolds were immobilized onto glass coverslips with an inert adhesive (Aquarium Sealant, King British, Gainsbrough, UK) that was allowed to cure overnight on the bench. The scaffolds were then incubated in 0.2% (w/v) tween 20/0.2% (w/v) triton X in PBS for 60 min before being washed in Milli‐Q H_2_O and the excess blotted away with filter paper. To label the cRGDS moiety, an amino‐Cy5 dye **7** was synthesized (Supporting Information), diluted to 0.1 × 10^−3^ m in 20 × 10^−3^ m sodium borate buffer solution at pH 9, and combined with 1‐ethyl‐3(3‐dimethylaminopropyl)carbodiimide (EDC, 2 × 10^−3^ m) and *N*‐hydroxysuccinimide (NHS, 2 × 10^−3^ m). This solution was applied to the immobilized scaffolds and the reaction proceeded on the bench at room temperature for 30 min before the immobilized scaffolds were washed with 20 × 10^−3^ m sodium borate buffer, Milli‐Q H_2_O, 0.2% (w/v) tween 20/0.2% (w/v) triton X solution, Milli‐Q H_2_O, 50% (v/v) IPA, 100% IPA, 50% (v/v) IPA, and further Milli‐Q H_2_O. The scaffolds were then blocked and labeled with fluorescein‐streptavidin as previously described, light protected, and imaged immediately. Sections were imaged on an upright Olympus BX51 epifluorescent microscope equipped with an Olympus DP70 camera. Images were obtained in three channels: Bright field, FITC to image the fluorescent brushes, and TxRed to image the Cy5 labeling of cRGDS.

*Cell Adhesion Assessment and Imaging of Dual Functionality Scaffolds*: Cell adhesion was assessed on the dual functionality scaffolds using a minor variation to the above protocol. Scaffolds and cells were prepared in an identical manner. When seeded, 25 000 cells were seeded on one side of the scaffold, incubated for 30 min to allow for cell attachment, gently turned over with sterile forceps, and a further 25 000 cells were seeded on the opposing side. The scaffold was then immobilized in a silicone‐coated well plate ensuring that there was free space for media below the scaffold (between the scaffold and the silicon coating). The plate was then returned to the incubator for 2 h for further cell attachment before NGM was gently added as per protocol. After 7 d the dual functional scaffolds were fixed in 4% (w/v) PFA and blocked in 1% (w/v) BSA and 0.1% (w/v) tween 20 as described above. 4′,6‐diamidino‐2‐phenylindole (DAPI, diluted 1:5000, Sigma) and phalloidin (diluted 1:200, Alexa Fluor 568, Invitrogen) were diluted in 1% (w/v) BSA solution and incubated with the scaffolds for 15 min. After washing in PBS the scaffolds were incubated in fluorescein‐streptavidin (diluted 1:500, Vector Laboratories) in PBS at pH 8.4 for a further 15 min. After washing in PBS the scaffolds were mounted on glass slides with Fluorosave mounting media (Calbiochem, VWR) and coverslipped. Slides were protected from light before being imaged on both the confocal and inverted optical microscopes as described above.

*Statistical Analysis*: All experimental test groups had a sample size of at least *n* = 3 for biochemical analysis. All cell‐related work was repeated with bovine tenocytes from three different animals. Data are presented as mean +/− standard deviation (SD). Statistical significance was determined by students T‐tests using Excel software, with a significance accepted where *p*‐value < 0.05.

## Supporting information

As a service to our authors and readers, this journal provides supporting information supplied by the authors. Such materials are peer reviewed and may be re‐organized for online delivery, but are not copy‐edited or typeset. Technical support issues arising from supporting information (other than missing files) should be addressed to the authors.

Supplementary
