# Supplementary material for: Modular and Versatile Spatial Functionalization of Tissue Engineering Scaffolds through Fiber‐Initiated Controlled Radical Polymerization
Source: Adv Funct Mater. 2015 Aug 17;25(36):5748–57. doi: 10.1002/adfm.201501277 (PMC4845664; doi:10.1002/adfm.201501277)
Supplement: Supplementary file 1 — Supplementary [file ADFM-25-5748-s001.pdf]

Copyright WILEY-VCH Verlag GmbH & Co. KGaA, 69469 Weinheim, Germany, 2015.

ADVANCED FUNCTIONAL MATERIALS

Supporting Information

for *Adv. Funct. Mater.*, DOI: 10.1002/adfm.201501277

Modular and Versatile Spatial Functionalization of Tissue Engineering Scaffolds through Fiber-Initiated Controlled Radical Polymerization

*Rachael H. Harrison, Joseph A. M. Steele, Robert Chapman, Adam J. Gormley, Lesley W. Chow, Muzamir M. Mahat, Lucia Podhorska, Robert G. Palgrave, David J. Payne, Shehan P. Hettiaratchy, Iain E. Dunlop, and Molly M. Stevens**

Supporting Information

Copyright WILEY-VCH Verlag GmbH & Co. KGaA, 69469 Weinheim, Germany, 2014

Supporting Information is available from the Wiley Online Library or from the author.

Modular and Versatile Spatial Functionalization of Tissue Engineering Scaffolds through Fiber-Initiated Controlled Radical Polymerization

*Rachael H. Harrison, Joseph A. M. Steele, Robert Chapman, Adam J. Gormley, Lesley W. Chow, Muzamir M. Mahat, Lucia Podhorska, Robert G. Palgrave, David J. Payne, Shehan P. Hettiaratchy, Iain E. Dunlop, Molly M. Stevens**

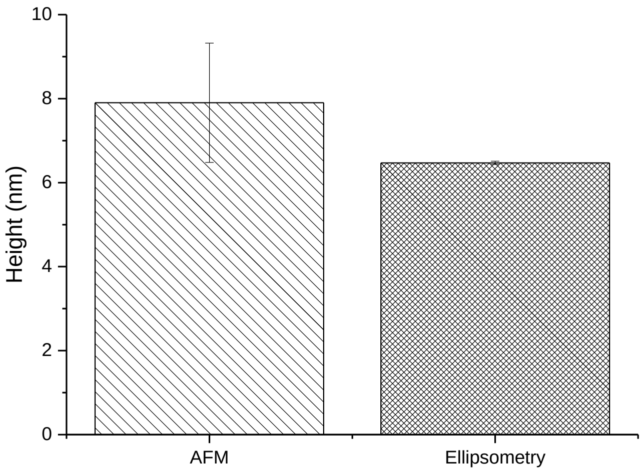

Figure S1. Height of dry brush measured by AFM (n = 3, error bars SD) and ellipsometry (n = 2, error bars SD) of pOEGMA grafted from 2D silicon surface. Theoretical polymer contour length = 24.7 nm.

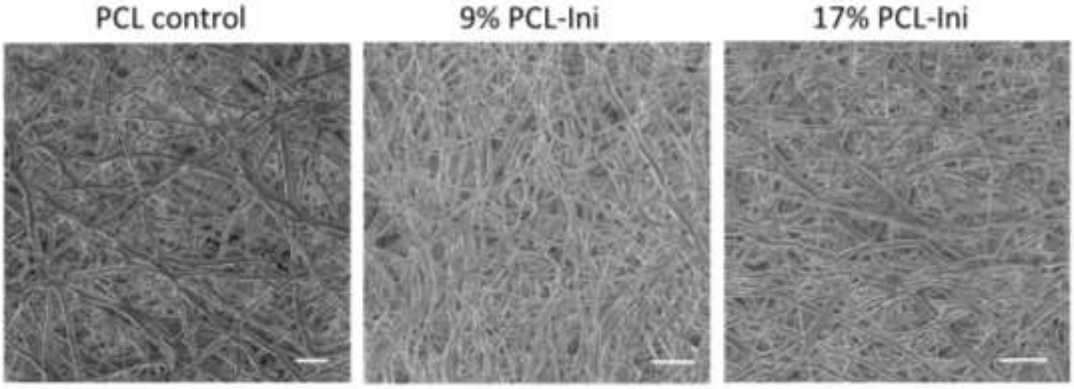

Figure S2. Scanning electron micrographs of plain PCL and modified PCL scaffolds with 9% (w/w) PCL-Ini and 17% (w/w) PCL-Ini. Scale bars 10µm.

XPS characterization of pOEGMA brush growth from 2D silicon and 3D electrospun surfaces

pOEGMA brushes that were grafted from 2D silicon wafers and 3D electrospun surfaces were analysed with XPS. The C-O bond is a good candidate for investigating the samples before and following functionalization with pOEGMA as the OEGMA is rich in these groups. High resolution spectra of the C1s peak obtained from all samples were found to be well fitted by 3 peaks: 285.0 eV (C-C or C-H), 286.5 eV (C-O single bond) and 288.4 eV (carboxyl or O=CO) [3,4] as shown in Figure 3, A. The C-O composition of the 2D and 3D PCL and PCL-pOEGMA scaffolds as obtained from the XPS spectra are presented in **Table S1**.

Table S1: C-O signal intensity of 2D silicon surfaces and 3D electrospun scaffolds as measured by XPS and the corresponding polymer characteristics

Repeat	+/- ATRP	2D silicon surfaces		3D electrospun scaffolds			Polymer characteristics			
		Si-APTES[a]	Si-APTES-Ini[a]	17% PCL[c]	9% PCL-Ini[b]	17% PCL-Ini[b]	X (%)	M_n (theo)[c]	M_n (SEC)[d]	$Đ$ (SEC)
**1**	-	12.2	11.6	12.0	15.5	12.9	-	-	-	-
**2**	-			15.9	9.5	13.6	-	-	-	-
**1**	+	23.9	59.4	19.6	27.3	38.2	78	56,200	44,800	1.12
**2**	+	15.8	56.8	8.4	34.5	33.9	79	56,900	45,000	1.13

Notes: [a] A single batch of silicon wafers was prepared and polymerized in two separate polymerization reactions, so surface 1 and 2 pre-reaction on the 2D silicon wafers are the same sample. [b] Duplicate mats were electrospun at each concentration of PCL and PCL-Ini. [c] Theoretical M_n calculated by ^{1}H-NMR. [d] SEC molecular weight estimated relative to polystyrene standards in DMF without correction to a universal calibration.

Table S2: C1s signal breakdown for the 2D silicon surfaces as measured by XPS

		C-C		C-O		O=CO	
**Surface replicate:**	**+/- ATRP**	**1**	**2**	**1**	**2**	**1**	**2**
Si-APTES	+	70.2	77.1	23.9	15.8	5.9	7.1
Si-APTES-Ini	+	33.6	34.5	59.4	56.8	7.0	8.7

Table S3: C1s signal breakdown for the 3D electrospun scaffolds as measured by XPS

		C-C		C-O		O=CO	
**Scaffold replicate:**	**+/- ATRP**	**1**	**2**	**1**	**2**	**1**	**2**
17% PCL	-	75.5	71.1	12.0	15.9	12.5	13.0
17% PCL-Ini	-	72.2	72.4	12.9	13.6	14.9	14.1
9% PCL-Ini	-	69.5	77.0	15.5	9.5	15.0	13.5
17% PCL	+	65.8	79.9	19.6	8.4	14.6	11.7
17% PCL-Ini	+	52.1	54.5	38.2	33.9	9.7	11.7
9% PCL-Ini	+	60.0	52.9	27.3	34.5	12.7	12.6

The ratio of oxygen content to the carbon increased upon PCL-pOEGMA grafting. This increment is due to the oxygen-containing groups (C-O and O=CO) within the pOEGMA surface coating. To further validate this, we quantify the details of the functionalization by peak fitting the C1s core spectra (Figure 3, A). A significant increase in C-O was found, which confirmed the presence of pOEGMA bound to PCL. The XPS data clearly demonstrates the success of pOEGMA functionalized on PCL mats.

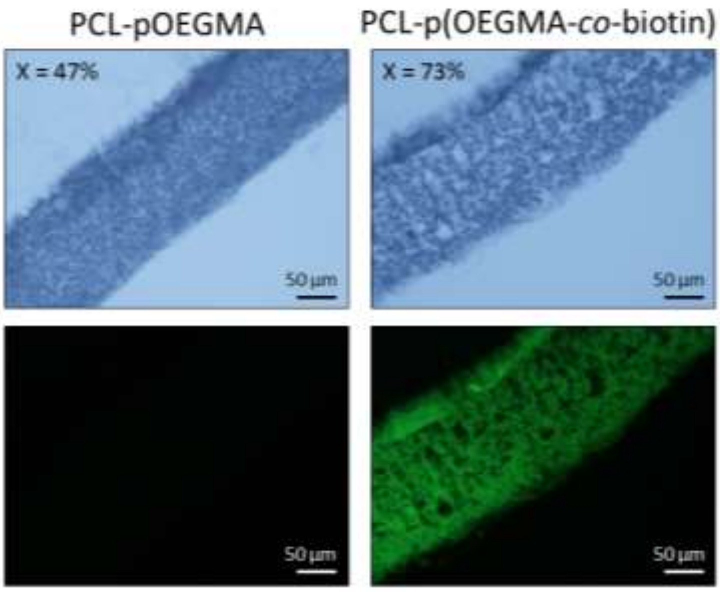

Figure S3. Fluorescent labelling and histological sectioning of PCL-pOEGMA and PCL-p(OEGMA-*co*-biotin) mats following incubation with streptavidin-fluorescein, demonstrating even distribution of polymer across the scaffold cross section. Electrospun mats of PCL-Ini 9% (w/w) underwent polymerization with OEGMA (left) and OEGMA and biotinylated PEG monomer (right) and were both subsequently labelled with fluorescein-

streptavidin, following which the scaffolds were embedded and histological sections were prepared. Bright field images (upper) and fluorescent images (lower) show signal on PCL-pOEGMA fibers and labelling of pOEGMA-*co*-biotin. Conversion of sacrificial initiator by ^{1}H-NMR (X) is inset.

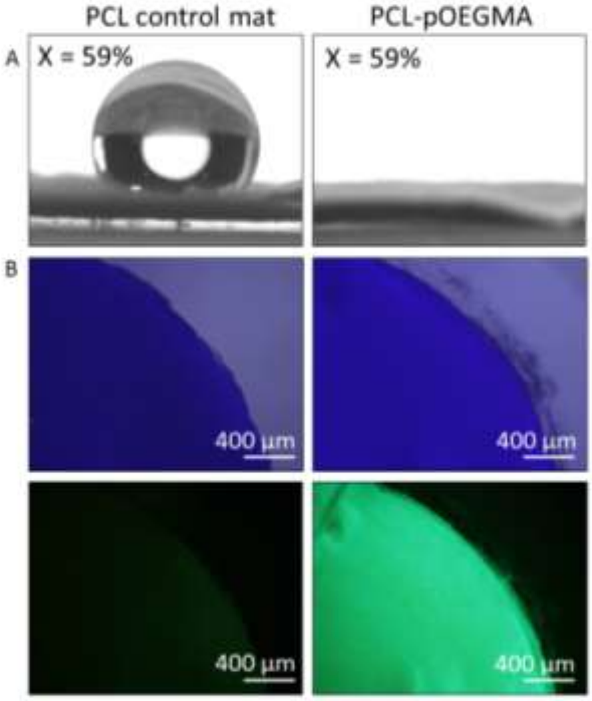

Figure S4. Confirmation of covalent attachment of pOEGMA to 3D electrospun scaffolds. (A) Water contact angle images of electrospun mats of control PCL (with no initiator) and PCL-Ini 17% (w/w) underwent polymerization with sacrificial initiator in solution. The control PCL remains hydrophobic following washing despite successful polymerization in solution and PCL-pOEGMA is highly hydrophilic suggesting no adsorption of pOEGMA onto fiber surfaces and successful covalent attachment when the initiating group (PCL-Ini) is present. (B) Electrospun mats of PCL and PCL-Ini 17% (w/w) underwent polymerization with OEGMA and biotinylated PEG monomer and subsequent fluorescein-streptavidin labelling. Bright field images (upper) and fluorescent images (lower) show minimal signal on control fibers and labelling of pOEGMA-*co*-biotin. Conversion of sacrificial initiator by ^{1}H-NMR (X) is inset.

*Synthesis of PCL-Ini **1***

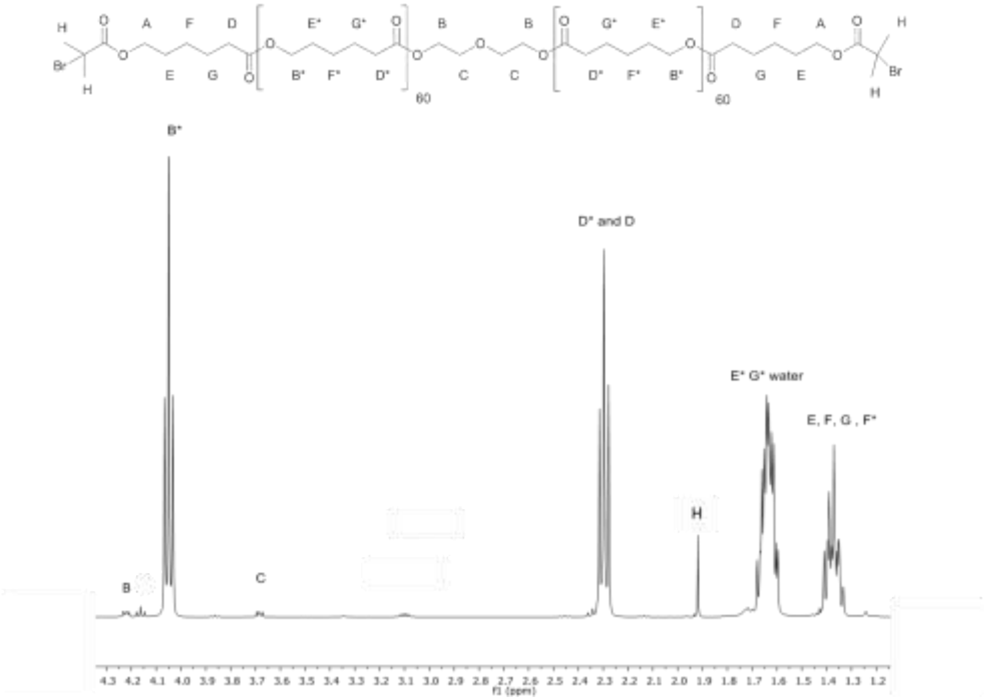

Figure S5. ^{1}H-NMR (400MHz, $CDCl_3$) of PCL-Ini 1

Synthesis of biotinylated PEG monomer 3 unit for fluorescent labelling of the polymer brush

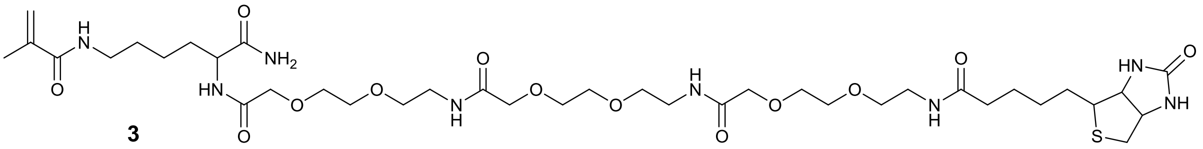

3

Figure S6. Structure of biotinylated PEG monomer 3

The biotinylated PEG monomer **3** was prepared using standard solid phase peptide synthesis (SPPS) techniques. Briefly, rink amide resin (0.7 mmol/g, 1 g) was deprotected and coupled to Fmoc-Lys(Mtt)-OH (4 eq., 2h). The Mtt group was then removed with 1.5% (v/v) TFA in DCM (2 x 15 min) and the resin washed with Hünigs base in DMF 1% (v/v) and DCM after which methacryloyl chloride (8 eq., 10 min) in DCM was coupled to the free amine. Three units of Fmoc-O2Oc-OH (2 eq., 3 h) were then coupled on resin, followed by a biotin group after the final fmoc deprotection. All couplings were performed with HBTU (4 eq.) and Hünigs base (8 eq.). The monomer was cleaved from the resin with a cocktail of TFA /

triisopropyl silane / H_2O (95 / 2.5 / 2.5 v/v/v, 2 h), triturated from diethyl ether and purified by HPLC (C18 column, H_2O / acetonitrile gradient). ESI-MS for $C_{38}H_{66}N_8O_{13}S$ Calcd. 875.5 $(M+H)^+$, Found 875.5. ^{1}H-NMR (400 MHz, $CDCl_3$) δ ppm: 8.08 – 7.90 (s, 1H, NH), 7.75 – 7.60 (m, 3H, NH), 7.59 – 7.37 (s, 2H, NH), 7.21 – 6.93 (m, 3H, NH), 6.70 – 6.55 (m, 2H, NH), 6.44 – 6.33 (m, 2H, NH), 5.75 (s, 1H, $\underline{H_2}C=C$), 5.41 (s, 1H, $\underline{H_2}C=C$), 4.8 – 4.5 (m, 2H, biotin), 4.45 (m, 1H, α-Lys), 4.10 (s, 6H, -HNCO-C$\underline{H_2}$-O-), 3.91 – 3.44 (m, 26H, O-C$\underline{H_2}$-C$\underline{H_2}$-, -$\underline{H_2}$C-NHCO-), 3.43 – 3.29 (m, 1H, biotin), 3.28 – 3.17 (m, 2H, biotin), 2.43 – 2.27 (m, 2H, biotin), 1.99 (s, 3H, C$\underline{H_3}$-C=CH$_2$), 1.95 – 1.36 (m, 12H, biotin and Lys CH$_2$).

Fluorescently labelled proteins

Bovine serum albumin (BSA) and fibronectin were fluorescently labelled by coupling to Rhodamine 6B isothiocyanate and NHS-fluorescein respectively. The BSA coupling was performed in PBS (10 mg/mL) and the fibronectin coupling was performed in borate buffer (20 mM, pH 9) at 1 mg/mL protein. In each case the dye (100 equiv) was added as a solution in DMSO (100 mM), and the solution was left stirring at RT for 2 hours. Both proteins were purified from the free dye by size exclusion chromatography over sephadex G100 in PBS.

Synthesis of PCL-cRGDS 2

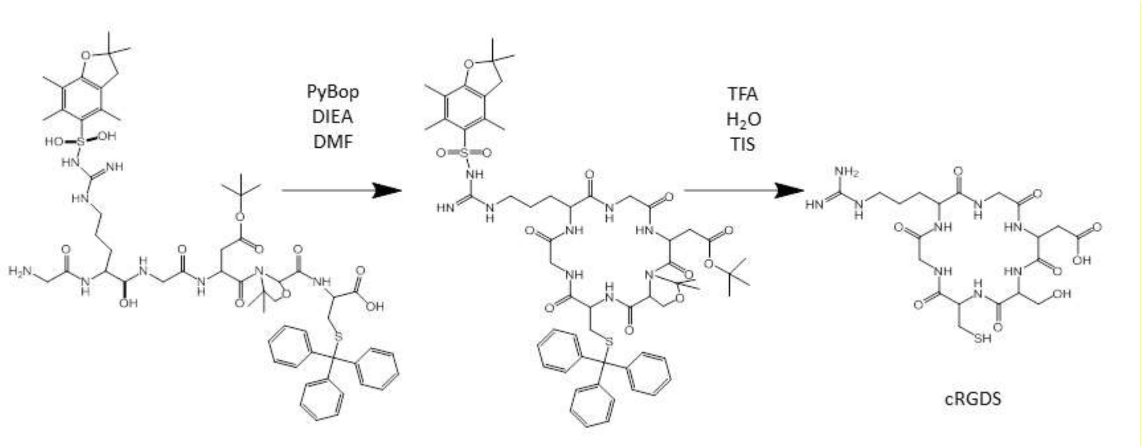

Figure S7. Synthesis scheme of cRGDS, adapted from Parmar *et al*. [5]

PCL-cRGDS was synthesized using a protocol adapted from the literature.[5-7] In brief, the cyclic RGDS peptide was synthesized on a 2-chlorotrityl chloride resin (100-200 mesh, VWR) at 1 mmol scale. Fmoc-Cys(Trt)-OH (Novabiochem) was dissolved in dichloromethane (DCM) at 1 molar equivalent and dimethylformamide (DMF) was added in small volumes until fully solubilized. Half of this solution was added to the resin along with 500 µL of *N*,*N*-diisopropylethylamine (DIEA), and the mixture was shaken on a wrist action shaker for 15 minutes. This process was repeated with the remaining solution followed by washing with DMF and DCM. A ninhydrin test was performed to assess the coupling reaction through the detection of free amines. Remaining free amines were capped by adding 5% (v/v) acetic anhydride (Sigma) with 2.5% (v/v) DIEA in DMF for 10 minutes two times with shaking. The resin was thoroughly washed with DCM and DMF before the ninhydrin test. After confirming there were no more unreacted amines, the Fmoc protecting group was removed using 20% (v/v) piperidine in DMF. The resin was washed with DCM and DMF, and Fmoc-Asp(Otbu)-Ser(psiMe, Mepro)-OH (Merck) was coupled at molar ratios of 2:1.95:3 [amino acid]:[HBTU]:[DIEA] in DMF. Remaining free amines were capped and all other amino acids were coupled as described previously.[5,6] The protected peptide was cleaved from the resin through the addition of 10 mL of 5% (v/v) trifluoroacetic acid (TFA) in DCM for 10 minutes with shaking. The solution was drained into a round bottom flask, and the resin washed with DCM until the solution was clear in the synthesis vessel. Excess DCM and TFA were removed with rotary evaporation to leave approximately 40 mL of solution. 10 mL ammonium hydroxide was added to neutralize the TFA followed by acetonitrile (ACN) to increase peptide solubility. The protected peptide was purified using reverse phase preparative high performance liquid chromatography (HPLC) running a mobile phase gradient of 80% ultrapure H_2O, 20% (v/v) ACN, and 0.1% (v/v) TFA. The solution was dried completely using rotary evaporation, and the product was re-dissolved in DMF at 1mg mL^{-1}.

The peptide was cyclized by adding 2 equivalents of benzotriazol-1-yl-oxytripyrrolidinophosphonium hexafluorophosphate (PyBop; AGTC Bioproducts) with 3 equivalents of DIEA overnight. The solvent was again removed by rotary evaporation and the remaining product dissolved in ACN/H_2O until solubilized. The cyclized peptide was purified by HPLC as previously described. The remaining protecting groups were removed using 95% (v/v) TFA with 2.5% (v/v) H_2O and 2.5% (v/v) triisopropylsilane (TIS). The unprotected peptide was precipitated in cold diethyl ether (DEE) and purified by HPLC. The final mass was confirmed using liquid chromatography-mass spectrometry (LC-MS) on an Agilent 6130 Quadrupole LC-MS coupled to an Agilent 1260 Infinity LC using a 150 x 4.6 mm Phenomenex Gemini NX C18 column with a 5 µm pore size and 100 Å particle size. The mobile phase consisted of ultrapure H_2O and ACN each supplemented with 0.1% (v/v) formic acid (VWR) by volume at a flow rate of 1 $ml.min^{-1}$. The peptide was eluted with a gradient of 95% (v/v) H_2O to 95% (v/v) ACN over 11 minutes. The electrospray source was operated with a capillary voltage of 3.2 kV and a cone voltage of 25 V with nitrogen used as the nebulizer and desolvation gas at a total flow of 600 L/h. The cRGDS did not stick to the column however electrospray ionization (ESI) of an early elution confirmed the correct mass.

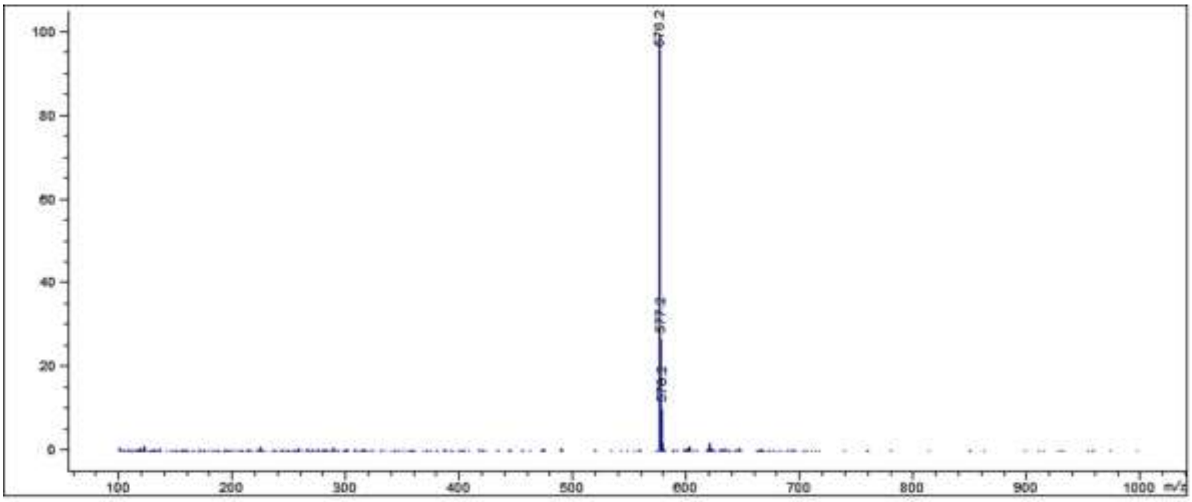

Figure S8. ESI of the cRGDS compound confirming correct mass of product (MW 577) which is from the same batch previously published in Parmar *et al*, Figure S3.[5]

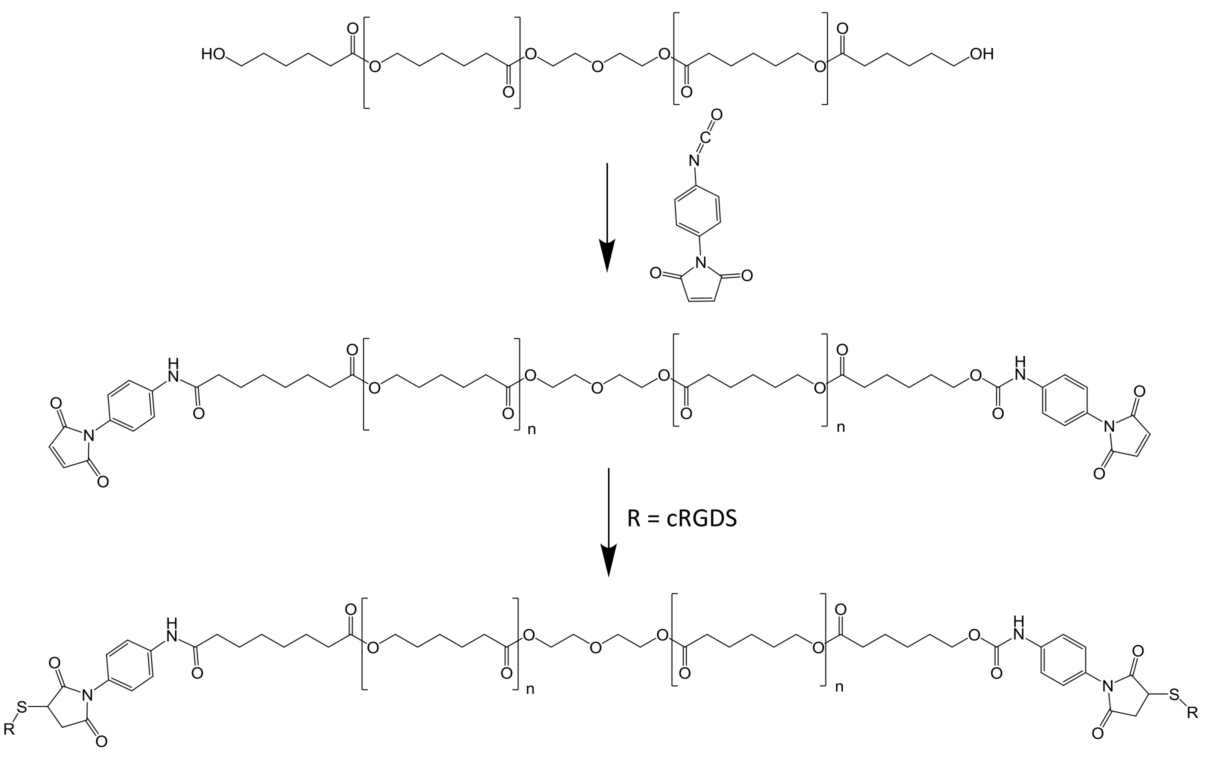

Figure S9. Synthesis scheme of PCL-cRGDS, adapted from Chow *et al.* [6]

Next the cRGDS was conjugated to the PCL using a protocol adapted from the literature. [6] PCL (Sigma, M_w 14 000, M_n 10 000) was dissolved in anhydrous n-methyl pyrrolidone (NMP, Sigma) under a nitrogen atmosphere. P-maleimidophenyl isocyanate (PMPI, Chem-

Impex International Inc.) at a 15 fold molar excess to PCL was also dissolved in anhydrous NMP and added drop-wise to the PCL solution while stirring and maintaining the nitrogen atmosphere. The reaction was allowed to proceed overnight and the product, PCL-maleimide, was precipitated into cold DEE and washed with H_2O to remove any unbound PMPI and dried under vacuum. The purified cRGDS peptide was dissolved in DMSO and the PCL-maleimide in anhydrous NMP in separate vessels. The cRGDS at 4 molar excess was added drop wise to the PCL-maleimide solution and left to react overnight with stirring under a nitrogen atmosphere. The resulting PCL-cRGDS conjugate was precipitated into cold DEE, washed with H_2O to remove any unreacted cRGDS, and dried in a vacuum dessicator prior to use. Synthesis steps of the solid product were confirmed by ^{1}H-NMR (400MHz Bruker spectrometer) and final conjugation to PCL was confirmed with Fourier transform infrared (FTIR) spectroscopy with a Perkin Elmer Spectrum One Spectrometer to evaluate the conjugation of the linker and cRGDS peptide to PCL. FTIR spectra were taken with a scanning wavenumber range from 4000 to 650 cm^{-1} and peaks were analyzed. The IR transmittance peaks at 3250 cm^{-1} and at 1630 cm^{-1} (amide bonds C=O, boxed) are present in the cRGDS pure peptide and the PCL-cRGDS, but not the control PCL (Figure S10) and demonstrate successful conjugation.

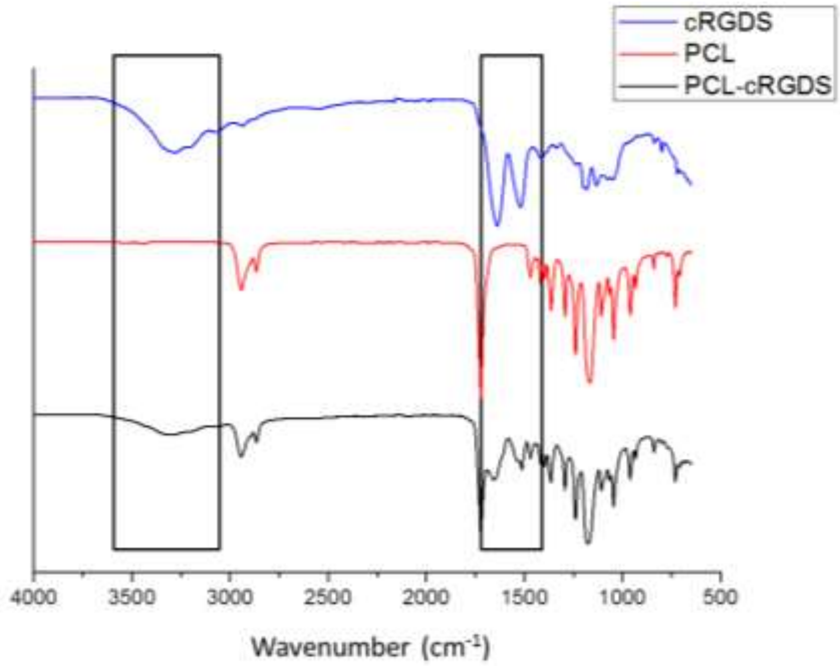

Figure S10: FTIR of the solid product demonstrating of successful conjugation of cRGDS to PCL.

Primary tenocyte cell culture and extraction

Bovine lower leg joints were purchased from an abattoir (C.E. Leech, Melborne, UK) following slaughter for the food industry. All cell consumables were purchased from Gibco® by Life Technologies and culture plastic purchased from Corning Inc. (USA) unless otherwise stated. Routine tissue culture was performed in normal growth media (NGM) and was made as follows. Dulbecco's Modified Eagle's Medium (DMEM)- GlutaMAX™ supplemented with 1 g/L D-glucose and pyruvate with 10% (v/v) Fetal Calf Serum (FBS), 1% (v/v) antibiotic/antimycotic (anti/anti; containing amphotericin B, streptomycin and penicillin). Cell work was performed in a category II laminar flow cell culture hood and the cells stored in an O_2/CO_2 incubator at 37°C, humidified with a CO_2 concentration of 5%.

The lower leg joints were refrigerated overnight before being cleaned with soap and water, then 70% (v/v) ethanol. Tendon extraction was performed using an aseptic technique and sterile instruments; the dorsal skin over the surface of the shin was opened and a supra-fascial

flap was raised to expose the extensor tendons. A length of tendon was excised en bloc and placed in a sterile petri dish primed with 15 mL of warmed NGM. The tendon pieces were macerated and the media aspirated. The tissue pieces were then immersed in 15 mL of 0.04% (w/v) collagenase type I in DMEM and 1% (v/v) anti/anti. The petri dishes were then returned to the incubator and agitated every few hours over a 24 hour period following which the fluid was passed through a 70 µm filter to remove undigested tissue. The cells were pelleted through centrifugation at 300 rcf at 37°C for 8 minutes following which they were re-suspended in media and counted using a haemocytometer. Cells were immediately seeded into a T25 cell culture flask primed with 5 mL of NGM at a density of 8.9x10^{4}/cm^{2} (the equivalent of 2M cells for a T225 flask). The cells were expanded to 80-90% confluence with media being exchanged twice weekly and then cryopreserved at passage 1 in DMEM, 20% (v/v) FBS and 10% (v/v) sterile dimethyl sulfoxide (DMSO) hybrid-max at ~10^{7}/mL in liquid nitrogen.

Prior to use in scaffold experiments, cells were removed from storage and seeded into T175 culture flasks primed with 25 mL NGM. Cells were expanded to 90% confluence before experimentation.

MTT assay and ladder for cell adhesion experiments

A stock solution of 5 mg mL^{-1} thiazolyl blue tetrazolium bromide (Sigma, UK) was prepared in sterile PBS and passed through a 0.22 µm syringe filter. Cell culture media was prepared using DMEM non-phenol red low glucose media supplemented with 1% (m/v) L-glutamine and used to dilute the bromide salt to a concentration of 1 mg mL^{-1} (MTT solution). The MTT solution was vortexed to ensure good mixing. The media was aspirated from the well plate and wells gently washed three times with warm sterile PBS to remove any debris and non-

adherent cells. 300 µL of the MTT solution was introduced to each well and the plate was incubated at 37°C for 2 hours after which time it was aspirated and the plate put on ice. 500 µL isopropanol (IPA) was added to each well and was agitated to encourage mixing. The plate was sealed and refrigerated to limit any IPA evaporation whilst the purple formazan crystals were solubilized. 100 µL of each well was removed in triplicate after thorough mixing with a pipette to a new 96 well plate. The plate was sealed, kept on ice and protected from light before absorbance being read at 550 nm on a SpectraMax M5 plate reader.

When performed on scaffolds, the MTT assay was performed as above with minor modification. Following aspiration of media and washing with PBS, the scaffolds were incubated with MTT solution as described above. After 2 hours, the scaffolds were gently removed from the well plate and put into 1.5 mL centrifuge tubes before 500 µL of IPA was added to the scaffolds. Scaffolds were vortexed within the tubes to facilitate the dissolution of all the formazan salt from any cells within the scaffolds. The remainder of the assay was performed as described above.

A cell ladder was created in a tissue culture treated 24-well plate with cells from the same passage. Serial dilutions were performed to give a ladder ranging from 100 000 cells to 3125 cells with cell free negative controls. Cells were cultured in the incubator for 2 hours until adhesion was observed, at which time a 3-(4,5-dimethylthiazol-2-yl)-2,5-diphenyltetrazolium bromide (MTT) cellular metabolism assay was performed.

*Synthesis of Amino Cy5 dye **7** for labelling of PCL-cRGDS (Figures S11-S13)*

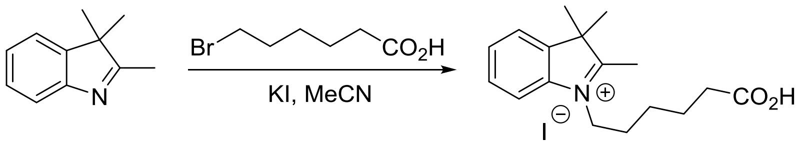

5

Figure S11. Synthesis scheme for compound 5.

Synthesis of compound 5 was adapted from Simmons *et al*.[8] 2,3,3-Trimethylindolenine (2.5 g, 15.5 mmol), 6-bromohexanoic acid (3.9 g, 20.2 mmol) and potassium iodide (3.4 g, 20.2 mmol) were heated to 85 ˚C in acetonitrile (30 mL) for 30 hours. After cooling to room temperature, the mixture was filtered and concentrated *in vacuo*. The residue was diluted with diethyl ether (300 mL) and left to precipitate at 4 ˚C overnight. The resultant solid was collected by filtration, washed with diethyl ether (2 x 50 mL) and dried *in vacuo*. A yield of 4.8 g, 12.1 mmol (61 %) was obtained as a red solid. Spectroscopic data were consistent with those previously reported.[8] ^{1}H NMR (400 MHz, DMSO): δ = 7.96-8.00 (1H, m, H8), 7.85 (1H, dd, *J* = 5.9, 2.9 Hz, H5), 7.61-7.67 (2H, m, H6 and H7), 4.46 (2H, t, *J* = 7.7 Hz, -C$\underline{H}_2$Ar), 2.84 (3H, s, -C$\underline{H}_3$), 2.24 (2H, t, *J* = 7.2 Hz, -C$\underline{H}_2$$CO_2$H), 1.84 (2H, tt, J_1 = J_2 = 7.8 Hz, -C$\underline{H}_2$$CH_2$Ar), 1.49-1.63 (8H, m, -(C$\underline{H}_3$)$_2$ and –C$\underline{H}_2$$CH_2CO_2$H), 1.35-1.49 (2H, m, -C$\underline{H}_2$$CH_2CH_2$Ar) ppm;

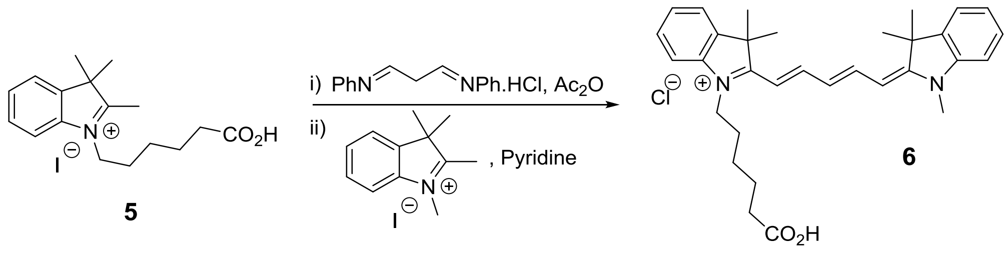

Figure S12. Synthesis of Cy5.HCL 6 from compound 5.

Synthesis of Cy5.HCl **6** was adapted from Kvach *et al*.[9] Indole **5** (2.4 g, 6 mmol) and malonaldehyde bis(phenylimine) monohydrochloride (1.8 g, 7.2 mmol) were dissolved in acetic anhydride (15 mL) and heated to 120 ˚C for 30 minutes. After cooling to room temperature 1,2,3,3-tetramethyl-3H-indolium iodide (2.5 g, 8.4 mmol) and pyridine (15 mL) were added and the reaction stirred for 18 hours during which time it turned dark blue. After concentration *in vacuo* at 80 ˚C, the residue was precipitated in hexane (100 mL), the solvent

decanted and the procedure repeated twice. The resultant blue oil was dissolved in chloroform (100 mL), washed with H_2O (2 x 100 mL) and brine (100 mL), dried with $MgSO_4$, filtered and concentrated *in vacuo*. The residue was purified by flash column chromatography, eluting with 10 % EtOH:$CHCl_3$. Pure fractions were concentrated *in vacuo* to give the DP as a purple foam. A yield of 2.5 g, 4.8 mmol (80 %) was obtained. Spectroscopic data were consistent with those previously reported.[9] ^{1}H NMR (400 MHz, DMSO): δ = 12.03 (1H, s, -COOH), 8.29-8.40 (2H, m, b-H), 7.52-7.67 (2H, m, $H5_{a/b}$), 7.36-7.53 (4H, m, $H7_{a/b}$ and $H8_{a/b}$), 7.20-7.34 (2H, m, $H6_{a/b}$), 6.51-6.63 (1H, m, c-H), 6.22-6.36 (2H, m, a-H), 4.09 (2H, t, *J* = 8.3 Hz, -CH_2Ar), 3.61 (3H, s, ArMe), 2.29 (2H, m, -CH_2CO_2H), 1.64-1.74 (14 H, m, -CH_2CH_2Ar and Ar$(CH_3)_2$), 1.50-1.60 (2H, m, -$CH_2CH_2CO_2H$), 1.39 (2H, dt, *J* = 15.3, 6.4 Hz, -$CH_2CH_2CH_2$Ar) ppm;

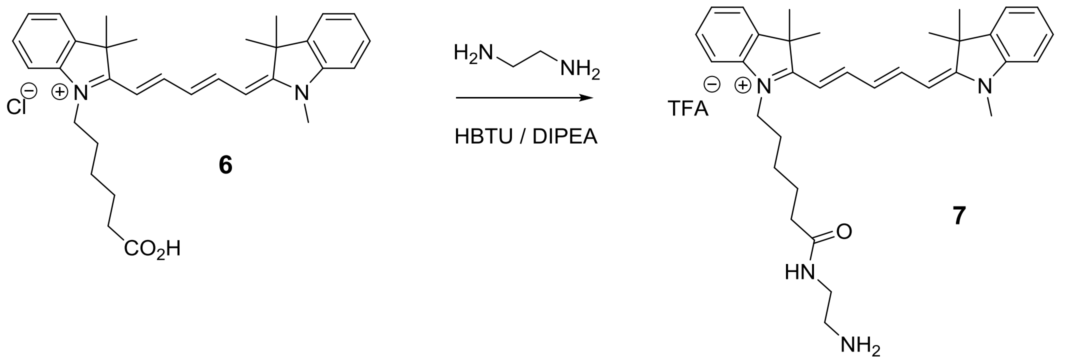

Figure S13. **Synthesis of amino Cy5 dye 7 from Cy5.HCL 6.**

Cy5.HCl **6** (65 mg, 0.12 mmol) and ethylene diamine (83 µl, 1.2 mmol) were then dissolved in dichloromethane (8 ml) and triethylamine (125 mg, 1.2 mmol) and HBTU (93 mg, 0.24 mmol) were added in DCM (2 ml). The reaction was stirred overnight at room temperature, concentrated, and precipitated twice from ice cold diethyl ether. The precipitate was purified by HPLC over a C18 column (H_2O / acetonitrile gradient) and freeze dried to yield the product as a dark blue solid. ESI-MS for $C_{34}H_{45}N_4O^+$ Calcd. 525.4 M^+, Found 525.4. ^{1}H-NMR (400 MHz, CDCl3) δ ppm: 7.94 – 7.73 (td, *J* = 13.1, 4.8 Hz, 2H, b-H), 7.44 – 7.29 (m,

4H, $\underline{H}5_{a/b}$ and $\underline{H}7_{a/b}$), 7.29 – 7.17 (m, 2H, $\underline{H}8_{a/b}$), 7.15 – 7.02 (d, *J* = 7.9 Hz, 2H, $\underline{H}6_{a/b}$), 6.60 (t, *J* = 12.4 Hz, 1H, c-$\underline{H}$), 6.36 – 6.02 (dd, *J* = 20.1, 13.4 Hz, 2H, a-$\underline{H}$), 3.99 (t, *J* = 7.5 Hz, 2H, $C\underline{H}_2Ar$), 3.57 (s, 3H, Ar$\underline{Me}$), 3.14 (s, 2H, $-C\underline{H}_2CH_2NH_2$), 2.36 (m, 2H, $-C\underline{H}_2CO$), 1.93 – 1.75 (m, 2H, $-C\underline{H}_2CH_2Ar$), 1.51 – 1.36 (m, 2H, $-CH_2C\underline{H}_2NH_2$), 1.68 (s, 12H, $Ar(C\underline{H}_3)_2$), 1.59 – 1.53 (m, 2H, $-C\underline{H}_2CH_2CO-$), 1.36 – 1.11 (s, 2H, $C\underline{H}_2CH_2CH_2Ar$).

References

[1] F. J. Xu, L. Y. Liu, W. T. Yang, E. T. Kang, K. G. Neoh, *Biomacromolecules,* **2009**, *10*, 1665.
[2] S. Yuan, D. Wan, B. Liang, S. O. Pehkonen, Y. P. Ting, K. G. Neoh, E. T. Kang, *Langmuir*, **2011**, *27*, 2761.
[3] S. Yuan, G. Xiong, A. Roguin, S. Hin Teoh, C. Choong in *Advances in Biomaterials Science and Biomedical Applications*, (Ed. R. Pignatello), InTech, Croatia, **2013**, pp 178-205.
[4] A. Martins, E. D. Pinho, S. Faria, I. Pashkuleva, A. P. Marques, R. L. Reis, N. M. Neves, *Small,* **2009**, *5*, 1195.
[5] P. A. Parmar, L. W. Chow, J. St-Pierre, C. Horejs, Y. Y. Peng, J. A. Werkmeister, J. A. M. Ramshaw, M. M. Stevens, *Biomaterials,* **2015**, 54, 213.
[6] L. W. Chow, A. Armgarth, J. –P. St-Pierre, S. Bertazzo, C. Gentilini, C. Aurisicchio, S. D. McCullen, J. A. M. Steele, M. M. Stevens, *Adv. Healthcare Mater.,* **2014**, *3*, 1381.
[7] J. S. Davies, *J. Pept. Sci.,* **2003**, *9*, 471.
[8] R. L. Simmons, R. T. Yu, A. G. Myers, *J. Am. Chem. Soc.*, **2011**, *133*, 15870.
[9] M. V. Kvach, A. V. Ustinov, I. A. Stepanova, A. D. Malakhov, M. V. Skorobogatyi, V. V. Shmanai, V. A. Korshun, *Eur. J. Org. Chem.,* **2008**, 2107.
